# COVID-19 vaccination safety and associated health care utilization among adults with inflammatory bowel disease – a population-based self-controlled case series analysis

**DOI:** 10.1186/s12876-024-03273-0

**Published:** 2024-05-30

**Authors:** Jennifer J. Y. Lee, Sasha Bernatsky, Eric I. Benchimol, M. Ellen Kuenzig, Jeffrey C. Kwong, Qing Li, Jessica Widdifield

**Affiliations:** 1 ICES Central, 2075 Bayview Avenue, Toronto, ON M4N 3M5 Canada; 2 Department of Paediatrics, University of Toronto, Toronto, ON Canada; 3 Division of Rheumatology and Clinical Epidemiology, Department of Medicine, McGill University Health Centre, Montreal, QC Canada; 4 SickKids Inflammatory Bowel Disease Centre, Division of Gastroenterology, Hepatology and Nutrition, The Hospital for Sick Children, Toronto, ON Canada; 5 Child Health Evaluative Sciences, SickKids Research Institute, The Hospital for Sick Children, Toronto, ON Canada; 6 Dalla Lana School of Public Health, University of Toronto, Toronto, ON Canada; 7 Sunnybrook Research Institute, Toronto, ON Canada; 8 Public Health Ontario, Toronto, ON Canada; 9 Department of Family and Community Medicine, University of Toronto, Toronto, ON Canada; 10 Institute of Health Policy, Management and Evaluation, University of Toronto, Toronto, ON Canada

**Keywords:** COVID-19, Vaccine Safety, Inflammatory bowel disease

## Abstract

**Background and aims:**

There is an incomplete understanding of the full safety profiles of repeated COVID-19 vaccinations in patients with inflammatory bowel disease (IBD). Among individuals with IBD, we assessed whether COVID-19 vaccines were associated with serious adverse events of special interest (AESI) and health care utilization [all-cause hospitalizations, Emergency Department (ED) visits, gastroenterology visits, IBD-related visits].

**Methods:**

Using comprehensive administrative health data from Ontario, Canada, adults with IBD who received at least one COVID-19 vaccine from December 2020-January 2022 were included. Self-controlled case series analyses were conducted to evaluate the relative incidence rates of AESI and health care utilization outcomes across post-vaccination risk and control periods.

**Results:**

Among 88,407 IBD patients, 99.7% received mRNA vaccines and 75.9% received *≥* 3 doses. Relative to control periods, we did not detect an increase in AESI. IBD patients had fewer all-cause hospitalizations during post-vaccination risk periods. Patients experienced more all-cause ED visits after dose 2 [Relative Incidence (RI):1.08(95%CI:1.04–1.12)] but fewer visits after doses 3 [RI:0.85 (95%CI:0.81–0.90)] and 4 [RI:0.73 (95%CI:0.57–0.92)]. There was no increase in gastroenterologist visits or IBD-related health care utilization post-vaccination. There were fewer IBD-related hospitalizations after dose 1 [RI:0.84 (95%CI:0.72–0.98)] and 3 [RI:0.63 (95%CI:0.52–0.76)], fewer IBD-related ED visits after dose 3 [RI:0.81 (95%CI:0.71–0.91)] and 4 [RI:0.55 (95%CI:0.32–0.96)], and fewer outpatient visits after dose 2 [RI:0.91 (95%CI:0.90–0.93)] and 3 [RI:0.87 (95%CI:0.86–0.89)].

**Conclusion:**

This population-based study did not detect increased AESI, all-cause or IBD-related health care utilization following COVID-19 vaccination, suggesting a lack of association between vaccination and increased disease activity.

**Supplementary Information:**

The online version contains supplementary material available at 10.1186/s12876-024-03273-0.

## Introduction

People living with inflammatory bowel disease (IBD) frequently use biologics, immunomodulators and systemic corticosteroids, which may increase risk of infections [1,2]. Having severe active inflammation [3] and using systemic corticosteroids [4] is associated with severe COVID-19 (SARS-CoV-2) infection. In addition, IBD patients on specific biologics and immunomodulators may require more frequent COVID-19 vaccinations in order to maintain a sufficient immune response [3,4]. Therefore, the safety of COVID-19 vaccines in the IBD patient population is an important concern.

A recent systematic review found that COVID-19 vaccines appear safe with only mild adverse events among individuals with IBD and flares to be infrequent [5]. However, most of the studies included in this review were small (thus unlikely to detect rare adverse events associated with vaccination) and uncontrolled. Study design is important, since studies simply comparing vaccinated and unvaccinated IBD individuals may be subject to selection and confounding biases. Employing a self-controlled case series (SCCS) design can overcome these concerns. By comparing outcomes temporally and by using individuals as their own controls, several selection and other biases are avoided. In addition, when a vaccine is administered in multiple doses, multiple risk and control periods can be assessed while controlling for clustering, thereby increasing efficiency [6].

We used population-based health administrative data from all publicly insured residents of Ontario, Canada (> 99% of the population of 14.4 million people). We assessed adverse events of special interest (AESI) in IBD individuals after COVID-19 vaccination, and also compared these rates to matched non-IBD controls. In addition, we compared emergency department (ED) visits, hospitalizations, gastroenterologist visits and IBD-related healthcare utilization before and after vaccination.

## Materials and methods

### Study design

We constructed population-based cohorts of adults with and without IBD, who received at least one COVID-19 vaccine from December 15, 2020 until January 16, 2022. A self-controlled case series analysis (SCCS) was performed with each cohort. A SCCS is an epidemiological study design that can investigate the association between a transient exposure (such as COVID vaccinations) and outcomes, by partitioning out a participant’s observation period to risk and exposure periods and then comparing outcome rates within individuals [7].

### Setting and data sources

This study was conducted in Ontario, Canada where information on all contacts with the health care system for all residents with universal single-payer healthcare (> 99% of the population) are captured within health administrative data. The data used in this study were analyzed at ICES using unique encoded identifiers, which permits deterministic linkage across all health administrative datasets.

The Registered Persons Database (RPDB) was used to identify inclusion criteria and to describe patient demographic characteristics. COVID-19 vaccination status (product, date administered, and dose number) were ascertained from the COVaxON database. To identify outcomes and co-morbidities, we used physician claim diagnosis codes from the Ontario Health Insurance Plan (OHIP) and identified hospital discharge diagnosis codes from the Canadian Institute for Health Information’s Discharge Abstract Database and ED visits from the National Ambulatory Care Reporting System (Supplementary Table S1). Gastroenterology visits were identified using the OHIP billing claims database. Information on prior SARS-CoV-2 infections was obtained from the C19INTGR database, which includes all Ontario SARS-CoV-2 PCR test results, but not home antigen test results. Prior influenza vaccination administered in physician offices and pharmacies was ascertained from the OHIP billing claims database and the Ontario Drug Benefit database, respectively.

The use of data in this study was authorized under Sect. 45 of Ontario’s Personal Health Information Protection Act, which does not require review by a Research Ethics Board. This study was approved by a privacy impact assessment at ICES (www.ices.on.ca).

### Study population

We used the Ontario Crohn’s and Colitis Cohort, which uses one of three validated administrative data case definitions to identify patients with IBD, based on age at the time of health care contact [pediatrics (< 18 years old), adults (18–64 years old), and elderly (*≥* 65 years old); Refer to Supplementary Table S1]. For example, an adult patient (between 18 and 64 years old) would require 5 outpatient visits or hospitalizations associated with the IBD diagnosis code for diagnosis. The sensitivity, specificity, positive predictive value, and negative predictive values of the three case definition algorithms ranged from 59 to 90%, 96–99%, 60–80%, and 95–99%, respectively [8,9].

In this study, patients with IBD were included if they were at least 18 years or older at the time of their first COVID-19 vaccine dose. A separate general population cohort was sampled from the RPDB, where four non-IBD comparators were matched on sex, age (± 2 years), and region of residence for each IBD patient. All individuals were required to have received at least one COVID-19 vaccine from December 15, 2020 until January 16, 2022. We excluded long-term care residents (who are frail and their threshold for hospitalization differs), and individuals who received out-of-province vaccines. All individuals were required to be actively enrolled with OHIP on June 14, 2020 (6 months prior to the start of the COVID-19 Vaccination Program in Ontario).

### Patient characteristics

Characteristics included patient age, sex, neighbourhood income quintile (discerned based on the individual’s residing postal code and census neighborhood income, with the 5th quintile representing the highest income), rural residence, prior history of SARS-CoV-2 infection and influenza vaccination, co-morbidities, and COVID-19 vaccine characteristics. Co-morbidities (which pre-dated COVID-19 vaccine exposure) included whether or not patients had a history of hypertension, chronic respiratory disease, diabetes, chronic heart disease, chronic kidney disease, advanced liver disease, dementia, stroke or transient ischemic attack and frailty (Refer to Supplementary Table S1 for definitions).

### Outcomes

AESI were treated as a composite outcome and were defined as a hospitalization or ED visit with a diagnosis code for the conditions of interest which included Bell’s palsy, idiopathic thrombocytopenia, acute disseminated encephalomyelitis, myocarditis, pericarditis, Guillain-Barre syndrome, transverse myelitis, acute myocardial infarction, anaphylaxis, stroke, deep vein thrombosis, pulmonary embolism, narcolepsy, appendicitis, and disseminated intravascular coagulation. These AESI were identified based on safety surveillance reports for COVID-19 vaccines [10–12]. The decision to group the AESI as a composite outcome was made a priori given that we suspected few events for each of these AESI had they been considered separately. As Bell’s palsy may be managed in ambulatory care settings, physician billing claims were also used to identify this condition. Diagnosis codes to ascertain these conditions are detailed in Supplementary Table S1. Event dates were defined according to admission dates (as opposed to discharge dates) and date of outpatient physician visit.

Secondary outcomes included all-cause hospitalizations, all-cause ED visits, gastroenterologist visits, and IBD-related health services. IBD-related health services were divided into IBD-related hospitalizations, IBD-related ED visits, and any IBD-related outpatient visit. IBD-related visits were defined by either a diagnostic code specific for IBD, or codes that were related to the signs, symptoms, and extra-intestinal manifestations of IBD, derived from expert opinion and consensus [13–15] (Supplementary Table S2).

### Risk and control periods

**Fig. 1:**
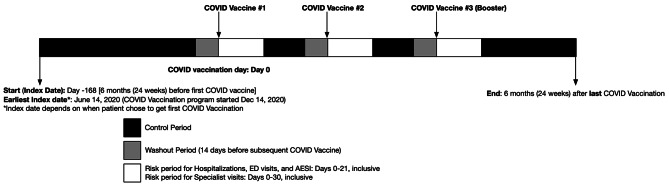
Description of risk and control periods for SCCS analysis In this example, a patient with IBD received a COVID-19 vaccine three times in the follow-up period. The risk and control periods were similarly derived for non-IBD comparators

The SCCS design requires the partitioning of an individual’s observation period into control and post-vaccination risk periods to compare the incidence of events within risk and control periods (Fig. 1). The pre-vaccination baseline control period was defined as the 6 months prior to the first COVID-19 dose but exclusive of the 14 days prior to the first dose (washout period) given concerns around the ‘healthy vaccinee effect’ (e.g. patients may choose to wait until they are in relatively good health before receiving a vaccine). Control periods in between doses started on day 22 from the last dose and ended 14 days prior to the next dose. A final control period commenced after the final dose risk period (up to a maximum of 6 months). AESI, hospitalizations and ED visits all required a 21-day risk period (the minimum time interval allowed before further COVID-19 vaccination doses) and a sensitivity analysis was performed to extend the risk period up to 42 days. If there were overlapping periods with subsequent vaccinations given < 42 days apart, the risk period would terminate the day before the next COVID-19 vaccine and the washout period is removed. For gastroenterologist or IBD-related outpatient visits, a 30-day risk period was used for the primary analysis, with an alternate 3-month (90 day) risk window used in the sensitivity analysis. This risk period after exposure is based on the concept that an IBD patient may flare after exposure but may experience delays in accessing their gastroenterologist due to pandemic-related restrictions.

### Analysis

Descriptive analyses were used to characterize individuals within each cohort. Standardized differences between the two groups were determined with a standardized difference larger than 0.10 indicating a clinically meaningful difference [16]. The risk (rate) of events were ascertained across control and risk periods. Poisson regression was used to estimate a crude relative incidence (RI), defined as the ratio of the incidence rate in the risk period to the incidence rate in the control periods. Relative incidence rate ratios (RIR) in each risk period vs. control period were estimated using non-IBD comparators. In our subgroup analyses, the relative incidence of AESI and health service utilization were reported by age and sex subgroups. Sensitivity analyses on extended risk windows (extended to 42 days for AESI, hospitalizations and ED visit events and 3-months for all-cause gastroenterologist or IBD-related outpatient visits) were additionally performed. Analyses were conducted using SAS Enterprise Guide 7.15 (SAS Institute Inc., Cary, NC).

## Results

### Cohort characteristics

The characteristics of the 88,407 adults with IBD and 353,628 age/sex matched non-IBD comparators who received at least 1 COVID-19 vaccine are presented in Table 1. The vast majority [88,175 (99.7%) of IBD patients and 352,342 (99.6%) of comparators] received at least 1 mRNA vaccine dose. Amongst individuals with IBD, 75.9% received *≥* 3 COVID-19 vaccines. Only 4,313 (4.9%) individuals with IBD and 2,862 (0.8%) individuals without IBD received more than 3 doses during the study period. The IBD cohort had a higher proportion of individuals with chronic kidney disease [IBD: 3,859 (4.4%) vs. non-IBD: 9,160 (2.6%), standard deviation, SD:0.10] and chronic respiratory disease [IBD: 24,843 (28.1%) vs. non-IBD: 75,930 (21.5%), SD:0.15]. More individuals with IBD received an influenza vaccine in the past 2 years relative to their comparators [IBD: 35,317 (39.9%) vs. non-IBD: 112,845 (31.9%), SD:0.17]

**Table 1:** Patient characteristics at the time of index date^1^, *n* (%) unless otherwise stated

Patient Characteristics	IBD *n* = 88,407	Non-IBD *n* = 353,628	Standardized difference
Median age at first vaccine dose (IQR)	54.0 (41.0–65.0)	54.0 (41.0–65.0)	0.00
Median IBD disease duration (IQR)	13.4 (6.9–22.6)	--	
Female	46,212 (52.3%)	184,848 (52.3%)	0.00
Male	42,195 (47.7%)	168,780 (47.7%)	0.00
Neighborhood income quintile			
1 (lowest)	13,492 (15.3%)	63,799 (18.0%)	0.07
2	16,565 (18.7%)	68,058 (19.3%)	0.01
3	18,142 (20.5%)	72,015 (20.4%)	0.00
4	18,918 (21.4%)	73,718 (20.9%)	0.01
5 (highest)	21,264 (24.1%)	75,907 (21.5%)	0.06
Rural residence	10,244 (11.6%)	42,780 (12.1%)	0.02
Hypertension	27,687 (31.3%)	109,206 (30.9%)	0.01
Chronic respiratory disease	24,843 (28.1%)	75,930 (21.5%)	0.15
Diabetes	12,430 (14.1%)	51,856 (14.7%)	0.02
Chronic heart disease	6,701 (7.6%)	22,343 (6.3%)	0.05
Chronic kidney disease	3,859 (4.4%)	9,160 (2.6%)	0.10
Frail	2,700 (3.1%)	7,510 (2.1%)	0.06
Prior stroke or transient ischemic attack	2,085 (2.4%)	7,122 (2.0%)	0.02
Dementia	999 (1.1%)	3,404 (1.0%)	0.02
Advanced liver disease	1,136 (1.3%)	1,545 (0.4%)	0.09
Prior SARS-COV-2 infection	479 (0.5%)	2,778 (0.8%)	0.03
Receipt of influenza vaccination in past 2 yrs	35,317 (39.9%)	112,845 (31.9%)	0.17
Number of COVID-19 vaccine doses during follow-up			
1	1,013 (1.1%)	4,995 (1.4%)	0.02
2	20,295 (23.0%)	104,764 (29.6%)	0.15
3	62,786 (71.0%)	241,007 (68.2%)	0.06
>3	4,313 (4.9%)	2,862 (0.8%)	0.25
At least 1 mRNA dose (BNT162b2 or mRNA-1273)	88,175 (99.7%)	352,342 (99.6%)	0.02
At least 1 BNT162b2 dose	71,997 (81.4%)	284,224 (80.4%)	0.03
At least 1 mRNA-1273 dose	42,649 (48.2%)	172,937 (48.9%)	0.01
At least 1 ChAdOx1 nCoV-19 dose	8,085 (9.1%)	32,564 (9.2%)	0.00

^1^Index date is 6 months (168 days) prior to an individual’s first vaccine dose

### Adverse events of special interest (AESI)

Relative to control periods, we did not detect a relative increase in AESI within 21 days of a COVID-19 vaccine in either IBD or comparator cohorts (Table 2). This appeared consistent regardless of the number of COVID-19 doses. Both IBD and comparator cohorts had reduced AESI risk after the 3rd dose [IBD RI: 0.65 (95% CI: 0.48–0.88), General Population RI: 0.76 (95% CI: 0.64–0.90)]. There was no statistically significant difference in the relative incidence rates when comparing AESI risk between the two groups after each dose.

**Table 2:** Crude rates and relative incidence rate ratio (RIR) of Adverse Events of Special Interest (AESI), All-cause hospitalizations, Emergency Department (ED) visits and gastroenterologist visits in the Postvaccine Risk Periods Referent to Control Periods in Adults with IBD and Non-IBD Matched Comparators

Outcome	Exposure/Risk Period	IBD		General Population Comparators		Relative Incidence Rate (RIR) Ratios^1^ between IBD and Comparators (95% CI)
		Number of Events	Relative Incidence (RI) Rate (95% CI)	Number of Events	Relative Incidence (RI) Rate (95% CI)	
Adverse Event of Special Interest [within 21 days of dose]	Control period	1833	Ref	5516	Ref	-
	Risk period after 1st dose	85	0.81 (0.64–1.03)	336	1.05 (0.92–1.19)	0.77 (0.59–1.01)
	Risk period after 2nd dose	112	1.09 (0.87–1.35)	329	1.05 (0.93–1.19)	1.04 (0.81–1.33)
	Risk period after 3rd dose	50	0.65 (0.48–0.88)	164	0.76 (0.64–0.90)	0.85 (0.60–1.21)
	Risk period after 4th dose	< 6	0.42 (0.10–1.81)	6	1.95 (0.56–6.77)	0.21 (0.03–1.45)
All-cause Hospitalization [within 21 days of dose]	Control period	14,462	Ref	29,571	Ref	-
	Risk period after 1st dose	676	0.81 (0.75–0.88)	1465	0.85 (0.80–0.90)	0.96 (0.87–1.06)
	Risk period after 2nd dose	705	0.87 (0.81–0.94)	1434	0.86 (0.81–0.91)	1.02 (0.92–1.12)
	Risk period after 3rd dose	460	0.77 (0.70–0.84)	922	0.80 (0.75–0.86)	0.95 (0.85–1.07)
	Risk period after 4th dose	22	0.46 (0.25–0.82)	45	1.69 (0.87–3.30)	0.29 (0.12–0.70)
All-cause ED visit [within 21 days of dose]	Control period	55,296	Ref	143,601	Ref	-
	Risk period after 1st dose	3196	1.00 (0.96–1.04)	8692	1.02 (1.00-1.05)	0.97 (0.92–1.02)
	Risk period after 2nd dose	3381	1.08 (1.04–1.12)	9055	1.10 (1.07–1.13)	0.98 (0.93–1.03)
	Risk period after 3rd dose	1936	0.85 (0.81–0.90)	4801	0.88 (0.85–0.91)	0.97 (0.92–1.03)
	Risk period after 4th dose	110	0.73 (0.57–0.92)	154	1.29 (0.87–1.92)	0.55 (0.35–0.87)

^1^RIR = Relative incidence rate ratios in each risk period between IBD and general population comparators

### All-cause ED visits and all-cause hospitalizations

When comparing post-COVID-19 vaccination periods to control periods, individuals with IBD had reduced all-cause hospitalizations after every COVID-19 dose, and this was statistically significant (Table 2). The relative incidence of hospitalization after dose 1 was 0.81 (95% CI: 0.75–0.88), dose 2 was 0.87 (95% CI: 0.81–0.94), dose 3 was 0.77 (95% CI: 0.70–0.84), and dose 4 was 0.46 (95% CI: 0.25–0.82). A reduced hospitalization rate post-COVID-19 vaccine was also observed in the comparator cohort as well, except for dose 4. Thus, the relative incidence rates were comparable after each COVID-19 dose other than after dose 4 where individuals with IBD experienced fewer hospitalizations than comparators [RIR: 0.29 (95% CI: 0.12–0.70)].

Individuals with IBD had equivalent risk for all-cause ED visits after dose 1 [RI: 1.00 (95% CI: 0.96–1.04)], an increase in risk after dose 2 [RI: 1.08 (95% CI: 1.04–1.12)], and reduced risk after doses 3 and 4 [Dose 3 RI: 0.85 (95% CI: 0.81–0.90), Dose 4 RI: 0.73 (95% CI: 0.57–0.92)] Among the comparator cohort, an increase in ED visits was observed after dose 2 [RI: 1.10 (95% CI: 1.07–1.13)] and a decrease in ED visits was observed after dose 3 [RI: 0.88 (95% CI: 0.85–0.91)] but not after dose 4 [RI: 1.29 (95% CI: 0.87–1.92)]. Hence, the relative incidence ratios were comparable between the cohorts except dose 4 where the IBD cohort had fewer ED visits than the comparator cohort [RIR: 0.55 (95% CI: 0.35–0.87)].

### IBD-related healthcare utilization

There was no increase in outpatient physician visits to gastroenterologists after vaccination. There was also no increase in any IBD-related health care visits post-vaccination (Table 3). Relative to control periods, individuals with IBD had fewer IBD-related hospitalizations after each dose, and this was significant after dose 1 [RI: 0.84 (95% CI: 0.72–0.98)] and dose 3 [RI: 0.63 (95% CI: 0.52–0.76)]. IBD-related ED visits were less likely to occur after dose 3 [RI: 0.81 (95% CI: 0.71–0.91)] and dose 4 [RI: 0.55 (95% CI: 0.32–0.96)]. IBD-related ED visits increased slightly after dose 2, but this was not statistically significant [RI: 1.07 (95% CI: 0.97–1.18)]. Overall, IBD-related outpatient visits were reduced after doses 2 and 3 [Dose 2 RI: 0.91 (95% CI: 0.90–0.93), Dose 3 RI: 0.87 (95% CI: 0.86–0.89)].

**Table 3:** IBD-Related Health Services Utilization in the Postvaccine Periods in Adults with IBD

Outcome	Exposure/Risk Period	Number of Events	Relative Incidence (RI) Rate (95% CI)
All-Cause Gastroenterologist visit [within 30 days]	Control period	14,875	Ref
	Risk period after 1st dose	1034	0.82 (0.77–0.88)
	Risk period after 2nd dose	1086	0.86 (0.81–0.93)
	Risk period after 3rd dose	851	0.89 (0.83–0.96)
	Risk period after 4th dose	79	1.21 (0.91–1.60)
IBD-Related Hospitalization [within 21 days of dose]	Control period	3754	Ref
	Risk period after 1st dose	181	0.84 (0.72–0.98)
	Risk period after 2nd dose	187	0.89 (0.77–1.03)
	Risk period after 3rd dose	97	0.63 (0.52–0.76)
	Risk period after 4th dose	7	0.57 (0.22–1.50)
IBD-Related ED Visits [within 21 days of dose]	Control period	8522	Ref
	Risk period after 1st dose	450	0.91 (0.82-1.00)
	Risk period after 2nd dose	521	1.07 (0.97–1.18)
	Risk period after 3rd dose	274	0.81 (0.71–0.91)
	Risk period after 4th dose	11	0.55 (0.32–0.96)
Any IBD-Related Outpatient Visit [within 30 days of dose]	Control period	251,795	Ref
	Risk period after 1st dose	21,211	1.01 (0.99–1.03)
	Risk period after 2nd dose	19,171	0.91 (0.90–0.93)
	Risk period after 3rd dose	13,986	0.87 (0.86–0.89)
	Risk period after 4th dose	1305	0.91 (0.83–1.01)

### Subgroup analyses

Sex-specific relative rates of AESI, all-cause hospitalizations, ED visits, and gastroenterology visits among individuals with IBD are shown in Table 4. All-cause ED visits were appreciably higher after the second dose regardless of age or sex. When stratified by age, individuals with IBD > 65 years old experienced fewer ED visits after dose 1 [RI: 0.89 (95% CI: 0.82–0.97)] and dose 3 [RI: 0.91 (95% CI: 0.83–0.99)], while individuals with IBD < 65 years old had fewer ED visits after dose 3 [RI: 0.82 (95% CI: 0.77–0.88)] and dose 4 [RI: 0.71 (95% CI: 0.53–0.96)]. Females with IBD had fewer ED visits after doses 3 [RI: 0.87 (95% CI: 0.81–0.93)] and 4 [RI: 0.60 (95% CI: 0.42–0.85)], while males with IBD had fewer ED visits after dose 3 [RI: 0.83 (95% CI: 0.77–0.90)].

**Table 4:** Age- and Sex-specific Relative Rates (with 95% CI) of AESI and Health Services Use among individuals with IBD, referent to control periods

Outcome	Exposure/Risk Period	Ages 18–65 Years	Ages > 65 Years	Females	Males
Adverse Event of Special Interest [within 21 days of dose]	Control period	Ref	Ref	Ref	Ref
	Risk period after 1st dose	0.93 (0.69–1.25)	- ^1^	0.93 (0.67–1.28)	0.70 (0.48-1.00)
	Risk period after 2nd dose	1.01 (0.75–1.36)	- ^1^	0.93 (0.67–1.28)	1.24 (0.92–1.66)
	Risk period after 3rd dose	0.50 (0.33–0.75)	- ^1^	0.63 (0.39–1.02)	0.66 (0.45–0.99)
	Risk period after 4th dose	0.83 (0.21–3.32)	- ^2^	0.40 (0.05–3.20)	0.46 (0.06–3.45)
All Cause Hospitalization [within 21 days of dose]	Control period	Ref	Ref	Ref	Ref
	Risk period after 1st dose	0.83 (0.76–0.92)	0.80 (0.69–0.92)	0.80 (0.72–0.90)	0.83 (0.73–0.93)
	Risk period after 2nd dose	0.85 (0.77–0.94)	0.92 (0.81–1.05)	0.78 (0.70–0.87)	0.98 (0.87–1.09)
	Risk period after 3rd dose	0.71 (0.63–0.81)	0.83 (0.72–0.95)	0.78 (0.69–0.88)	0.75 (0.66–0.87)
	Risk period after 4th dose	0.37 (0.15–0.92)	0.57 (0.29–1.12)	0.29 (0.11–0.81)	0.67 (0.33–1.36)
All Cause ED visit [within 21 days of dose]	Control period	Ref	Ref	Ref	Ref
	Risk period after 1st dose	1.04 (0.99–1.09)	0.89 (0.82–0.97)	1.03 (0.97–1.08)	0.96 (0.90–1.02)
	Risk period after 2nd dose	1.08 (1.03–1.13)	1.09 (1.02–1.17)	1.06 (1.01–1.12)	1.10 (1.03–1.17)
	Risk period after 3rd dose	0.82 (0.77–0.88)	0.91 (0.83–0.99)	0.87 (0.81–0.93)	0.83 (0.77–0.90)
	Risk period after 4th dose	0.71 (0.53–0.96)	0.76 (0.53–1.10)	0.60 (0.42–0.85)	0.90 (0.66–1.23)
Gastroenterologist Visit [within 30 days]	Control period	Ref	Ref	Ref	Ref
	Risk period after 1st dose	0.84 (0.78–0.91)	0.76 (0.66–0.89)	0.81 (0.74–0.89)	0.84 (0.76–0.92)
	Risk period after 2nd dose	0.85 (0.79–0.92)	0.91 (0.79–1.05)	0.86 (0.78–0.94)	0.87 (0.79–0.97)
	Risk period after 3rd dose	0.88 (0.80–0.96)	0.95 (0.81–1.10)	0.87 (0.78–0.96)	0.92 (0.82–1.04)
	Risk period after 4th dose	1.33 (0.97–1.82)	0.90 (0.48–1.66)	1.22 (0.85–1.77)	1.18 (0.77–1.82)

^1^Not able to estimate due sparse data and poor fit

^2^No events observed within the risk period

All-cause hospitalizations occurred less frequently after COVID-19 doses in females and in those < 65 years old. In individuals with IBD > 65 years old, hospitalizations were lower after dose 1 [RI: 0.80 (95% CI: 0.69–0.92)] and dose 3 [RI: 0.83 (95% CI: 0.72–0.95)]. In males with IBD, hospitalizations were appreciably lower only after dose 3 [RI: 0.75 (95% CI: 0.66–0.87)].

Relative to control periods, all-cause gastroenterologist visits decreased appreciably after the first 3 doses in individuals < 65 years old and in females. In individuals older than 65 years old, gastroenterology visits were less frequent only after dose 1 [RI: 0.76 (95% CI: 0.66–0.89)]. In males with IBD, gastroenterology visits were less frequent after dose 1 [RI: 0.84 (95% CI: 0.76–0.92)] and dose 2 [RI: 0.87 (95% CI: 0.79–0.97)].

### Sensitivity analyses

With the use of alternate risk windows (Supplementary Table S3), the only notable difference observed was that the rate of gastroenterology visits was appreciably lower only after dose 1 [RI: 0.89 (95% CI: 0.84–0.94)], while the rates of visits remained similar to control periods after doses 2 [RI: 0.96 (95% CI: 0.92-1.00)] and 3 [RI: 0.95 (95% CI: 0.91-1.00)], and were higher after dose 4 [RI: 1.41 (95% CI: 1.15–1.72)].

## Discussion

In this population-based self-controlled case series, we did not detect a significant increase in AESI when comparing risk and control periods among people living with IBD. Reassuringly, all-cause and IBD-related hospitalizations were either significantly lower or no different to control periods in individuals with IBD following every COVID-19 vaccine dose. Similarly, all-cause and IBD-related ED visits were reduced or no different to control periods with the exception of the second dose. Overall, these findings are an important addition to our knowledge supporting the safety of COVID-19 vaccines and provide reassurance for individuals living with IBD, which is particularly important given that vaccine safety, particularly concerns about vaccines causing a disease flare, may fuel vaccine hesitancy in this population [17–19].

The reassuring safety profile of COVID-19 vaccinations in the IBD population reported in our study is similar to other studies in the literature. A systematic review and meta-analysis of six IBD vaccine safety studies estimated that 2% experienced a severe adverse event and 1% of patients reported a disease flare following vaccination [5]. These estimates were largely influenced by two studies with unusually high rates of severe adverse events (33% and 9%) compared to the low percent (< 1%) in the other studies, which are more consistent with our own results [20,21]. One of the two studies, in particular, had a higher proportion of IBD patients with active disease (approximately two-thirds) at the time of study, which could have skewed the rate of adverse events as both an adverse event and IBD flare can precipitate an ED visit or hospitalization [21]. By conducting a population-based SCCS study design, the distribution of patients likely reflects a more realistic distribution of disease severity and accounted for unmeasured factors like IBD-related severity or activity (given that individuals serve as their own control) [22].

In individuals with IBD, we noted lower all-cause hospitalizations in all post-vaccine risk periods, and lower ED visits after doses 3 and 4. The explanation for these findings may be multifactorial. First, the lower health services use may be observed because of the protective effects of earlier COVID-19 vaccinations. With vaccine receipt, individuals may be at lower risk of SARS-CoV-2 infection (and hospitalization) which may also reduce the risk of post-viral IBD flare. Studies have demonstrated that between 7 and 10% of patients with IBD will experience an IBD flare within 3 months of a SARS-CoV-2 infection [23]. A decrease in IBD-related health service use has been similarly described in another SCCS study following influenza vaccination in children with IBD [24]. Second, it may also be possible that we observed lower rates in all post-vaccine risk periods because of the ‘healthy vaccinee’ effect, where individuals choose to be vaccinated when they feel well and when they feel their IBD control is best, thereby reducing health services use [25]. We attempted to account for the phenomenon in our study design by incorporating a washout period where the 2 weeks prior to the vaccine was excluded from our analyses. It is possible that the ‘healthy vaccinee’ effect can continue beyond the date of the vaccine. In some SCCS studies, a post-vaccination wash-out period is often incorporated. However, given that some of the AESI of interest are likely to be immediate after vaccination (i.e. anaphylaxis) and because we did not want to miss potentially important safety events, we decided a priori to exclude a post-vaccination wash-out period. Thus, it is possible that some residual ‘health vaccinee’ effect may have lowered our event rates.

Interestingly, our study demonstrated higher ED visit rates after the second dose in both IBD and general population comparators. It is unclear what is driving this increase in visits, but there may be the possibility that this increase is due to the vaccination exposure. Some studies have shown that certain AESI (such as myocarditis and pericarditis) and non-severe side effects are increased after sequential doses [26,27]. While our serious AESI rates did not detect a similar trend and our overall hospitalization rates were reduced, it may be possible that an increase in local or less severe side effects (such as expected local or systemic reactions like myalgias, fever, and headaches) after subsequent doses prompted more frequent ED visits. It is also possible that after two COVID-19 vaccinations (considered fully vaccinated in most individuals), individuals may have developed an increased sense of security and protection against the infection [28]. This may decrease self-isolation behaviours, prompting more ED visits unrelated to COVID-19 vaccinations (i.e. visits to the ER for trauma).

When the IBD cohort was divided according to sex and age, associations between COVID-19 vaccination and less health care use appeared more pronounced in those < 65 years old and in females. It may be possible that we did not see an appreciable difference in health care use in older adults with IBD as they may have other numerous comorbid conditions that increase their health care service use, irrespective of COVID-19 vaccine exposure. It may also be possible that older adults exhibit less severe adverse reactions to vaccination events in general due to immunosenescence [29,30].

Given that we could not directly ascertain inflammatory activity after COVID-19 vaccines from our administrative data due to the absence of detailed clinical information, we used gastroenterology visits and IBD-related health services utilization as proxies for disease flares. Our study did not detect an increase in either gastroenterology or IBD-related health services utilization following vaccination. Our findings appear to be consistent with other studies. One study from Israel comparing vaccinated and unvaccinated individuals with IBD found a higher risk of IBD flares among those who received COVID-19 vaccination (44% vs. 34%); however, this association was no longer significant when additionally matching based on the number of IBD flares in the two previous years [31]. Two other studies also suggested no clear change in IBD activity indices following COVID-19 vaccination [32,33]. A recent population-based study from the UK used a similar study design to ours to evaluate IBD flares post-COVID vaccination. IBD flares were defined as a primary care visit with a corticosteroid prescription, which is not typical of routine clinical practice in Canada (typically gastroenterologists will evaluate and manage IBD flares as opposed to primary care physicians). Similar to our study, that study reported no association between COVID-19 vaccinations and IBD flares [34].

This study has several strengths. Ontario has a universal, publicly-funded health care system and a centralized COVID-19 vaccine registry, and IBD patients are identified using a validated algorithm. This minimized any potential selection biases and misclassification of IBD status or vaccination exposure. By including a comparator group of age- and sex-matched controls, as well as SCCS analyses, we were able to compare vaccine safety both among people with IBD and relative to individuals without IBD. The SCCS design helps deal with unmeasured factors like disease severity (unavailable in administrative health data) and medications. In this cohort, the majority of IBD patients received three COVID-19 vaccines, generating sufficient sample sizes up to the third dose. These findings are reassuring, particularly given the current multi-dose vaccination strategy.

Our study may have some limitations, including the fact that diagnostic codes are not always clinically verified. To overcome this, we used a validated approach to identify individuals with IBD [8,9]. However, while we used AESI definitions that are in keeping with other population-based vaccine safety studies [35], not all of the specific adverse event definitions have been formally validated. As well, although SCCS designs take time-independent confounders into consideration, there may have been changes in a variety of time-dependent covariates such as disease status, drugs, SARS-CoV-2 activity levels within the community, and protective behaviours that we were not able to incorporate. Patients received their COVID-19 vaccines at different time periods upon vaccination roll out. Our analyses did not take into consideration external factors such as varying surges of SARS-CoV-2 activity and timing of various provincial lockdowns. As such, it is possible that the number of outpatient gastroenterology visits was underestimated. However, it is reassuring that we do not see a compensatory increase in ED visits due to potential lack of access with outpatient care.

Given the limitations of health administrative databases in Ontario, we did not have medication information for our entire patient cohort, and thus we are unable to identify if certain subgroups of IBD patients were at more risk of adverse events because of their medication history. However, prolonged medications that are unlikely to change (such as biologics which are typically administered for years as opposed to months) are unlikely to impact our results given the use of the SCCS design. The number of people receiving a fourth vaccine dose was limited and our comparisons of AESI and health services use following this dose may have been underpowered. Finally, we did not differentiate whether outcomes would be different between various mRNA vaccines and bivalent formulations were not yet available during the study period.

In conclusion, this large population-based study of individuals with IBD found no increased AESI, hospitalizations, or ED visits immediately following COVID-19 vaccination. These findings are an important addition to our knowledge supporting the safety of COVID-19 vaccines and provide reassurance for individuals living with IBD, which is particularly important given that concerns around vaccine safety, including disease flare, can fuel vaccine hesitancy in this population.

### Electronic supplementary material

Below is the link to the electronic supplementary material.

Supplementary Material 1

## Data Availability

The datasets used and/or analysed during the current study available from the corresponding author on reasonable request. Please note, the study dataset is held securely in coded form at ICES. While legal data sharing agreements between ICES and data providers (e.g., healthcare organizations and government) prohibit ICES from making the dataset publicly available, access might be granted to those who meet prespecified criteria for confidential access, available at www.ices.on.ca/DAS (email das@ices.on.ca). The full dataset creation plan and underlying analytic code are available from the corresponding author upon request, understanding that the computer programs might rely upon coding templates or macros that are unique to ICES and are therefore either inaccessible or require modification. The corresponding author affirms that the manuscript is an honest, accurate, and transparent account of the study being reported; that no important aspects of the study have been omitted; and that any discrepancies from the study as planned have been explained.

## Declarations

### Patient and public involvement

It was not possible to involve patients or the public in the design, or conduct, or reporting. Patient partners will be involved in knowledge dissemination.
