# Supplementary material for: COVID-19 vaccination safety and associated health care utilization among adults with inflammatory bowel disease – a population-based self-controlled case series analysis
Source: BMC Gastroenterol. 2024 May 30;24:189. doi: 10.1186/s12876-024-03273-0 (PMC11137996; doi:10.1186/s12876-024-03273-0)
Supplement: Supplementary file 1 — Supplementary Material 1 [file 12876_2024_3273_MOESM1_ESM.docx]

**LIST OF SUPPLEMENTARY TABLES**

Supplementary Table S1. Codes to define diagnoses/co-morbidities/covariates

Supplementary Table S2. OHIP, ICD-9, and ICD-10 codes used to identify IBD-related health services utilization

Supplementary Table S3. Sensitivity analyses of the SCCS method with risk windows extended to 42 days for serious AESIs, hospitalizations and ED visit events and 3-month risk window for gastroenterologist consultation visits after COVID-19 vaccination.

**Supplementary Table S1. Codes to define diagnoses/co-morbidities/covariates**

| **Diagnoses/Comorbidities/Covariates** | | |
| --- | --- | --- |
|  | **Database** | **Codes/Definitions** |
| IBD | OCCC | ICD-9: 555.x, 556.x  ICD-10: K50.x, K51  **Pediatric (<18 years old)**: If underwent colonoscopy, require 4 outpatient visits or 2 hospitalizations within 3 years. If did not undergo colonoscopy, require 7 outpatient visits or 3 hospitalizations within 3 years.  **Adult (18-64 years old):** 5 outpatient visits or hospitalizations within 4 years  **Elderly (>65 years old):** 5 outpatient visits or hospitalizations within 4 years and at least one prescription for an IBD-related medication |
| Long-term care residents | CCRS_LTC  ODB | Previously included in the CCRS_LTC database  ODB: Long Term care flag for any previous ODB claim |
| COVID-19 vaccination details | COVaxON database |  |
| Emergency Department utilization | NACRS | All cause (any ED visit) |
| Hospitalization | CIHI-DAD | All cause |
| Bell’s palsy | CIHI-DAD  NACRS  OHIP | ICD-10: G51.0  OHIP: 351 |
| Idiopathic thrombocytopenia | CIHI-DAD  NACRS | ICD-10: D69.3x, D69.4, D69.5, D69.6, D82.0, M31.1 |
| Acute disseminated encephalomyelitis | CIHI-DAD  NACRS | ICD-10: G04.0, G04.x, G05.x, G35, G36.x, G37.x, G92, G93.4, G96.9 |
| Myocarditis/pericarditis | CIHI-DAD  NACRS | ICD-10: I30.x, I32.x, I40.x, I41.x, I51.4, I40.x, I41.x, I51.4, I30.x, I32.x |
| Guillain-Barré syndrome | CIHI-DAD  NACRS | ICD-10: G61.0 |
| Transverse myelitis | CIHI-DAD  NACRS | ICD-10: G37.3, G36.0, G37.0, G37.8, G37.9 |
| Myocardial Infarction | CIHI-DAD  NACRS | ICD-10: I21.x, I22.x, I51.3 |
| Anaphylaxis | CIHI-DAD  NACRS | ICD-10: T78.2, T80.5, T78.2, T88.6 |
| Non-hemorrhagic and hemorrhagic stroke | CIHI-DAD  NACRS | ICD-10: I60.X, I61.X, I62.X, I63.X, I65.X, I66.X |
| Deep Vein Thrombosis | CIHI-DAD  NACRS | ICD-10: I82.X |
| Pulmonary Embolism | CIHI-DAD  NACRS | ICD-10: I26.X |
| Narcolepsy | CIHI-DAD  NACRS | ICD-10: G47.4 |
| Appendicitis | CIHI-DAD  NACRS | ICD-10: K35.X, K36.X, K37.X |
| Disseminated intravascular coagulation | CIHI-DAD  NACRS | ICD-10: D65.X |
| Prior SARS-CoV-2 infections confirmed with PCR testing | C19INTGR |  |
| Influenza vaccine received | OHIP  ODB | An OHIP billing with any of the following fee codes from October 1, 2019 to September 30, 2020, or October 1, 2020 up to 14 days before the index date: G590, G591, G592, Q130, Q590, Q690, Q691;  or, an ODB billing with any of the following Drug Identification Numbers (DINs)/Product Identification Number (PINs) from October 1, 2019 up to September 30, 2020: 02420643, 02420783, 02432730, ​​02473283, or October 1, 2020 up to 14 days before the index date: 02420643, 02420783, 02432730, 02445646, 02494248, 09857645, 09857646 |
| Household income quintile |  | Calculated at the dissemination area (DA) level using Census data by multiplying the median income (before-tax) by the number of households and dividing by the sum of single-person equivalent to obtain income per single person equivalent.  A DA is the smallest standard geographic area for which all census data are disseminated. A DA generally comprises approximately 400-700 people, but in densely populated cities may contain several thousand people.  We assigned subjects to a DA using postal code, as recorded in the Registered Persons Database.  For DAs where median income was unavailable, neighbouring DAs were used to estimate income per single person equivalent. DA-based income quintiles were constructed separately for each census metropolitan area or census agglomeration (one or more adjacent municipalities integrated via commuting flows). DAs within each such area were ranked from the lowest average income per single-  person equivalent to the highest, and DAs were assigned to five groups, such that each group contained approximately one-fifth the total in-scope population of each area. |
| Rurality | RIO | Urban-rural residential location was assessed using the Rurality index of Ontario (RIO).  Those with a RIO score of 0–39 were considered urban and those with a RIO of 40 and above were considered rural. |
| Patient demographics | RPDB |  |
| Chronic kidney disease | CIHI-DAD  OHIP | We defined this variable as having a CKD diagnosis code in DAD, NACRS, OHIP in the past 5 years, or: at least 1 dialysis code in each of the 3 months prior to index  OHIP: 403, 585  ICD-10: E102, E112, E132, E142, I12, I13, N08, N18, N19  OR  Patients who were on chronic dialysis in the year before index date, identified as those with at least 2 of any of the following codes in OHIP, DAD, or SDS separated by at least 90 days, but less than 150 days:  OHIP service codes: R849, G323, G325, G326, G860, G862, G865 G863, G866, G330, G331, G332, G333, G861, G082, G083, G085, G090, G091, G092, G093, G094, G095, G096, G294, G295, G864, H540, H740  DAD, SDS:  CCI procedure codes: 5195, 6698  CCP procedure code: 1PZ21 |
| Hypertension | HYPER database | ≥2 OHIP claims within two-years or ≥1 hospitalizations with a diagnosis code for hypertension which included ICD 10 codes (I10, I11, I12, I13, I15), ICD-9 codes (401, 402, 403, 404, 405), and OHIP codes (401, 402, 403, 404, 405) |
| Chronic heart disease | CIHI-DAD  OHIP | Individuals were defined as having “chronic heart disease” if they had congestive heart failure (CHF), ischemic heart disease, or atrial fibrillation. The definitions for these conditions are as follows:  CHF:  An ICES-derived CHF database was used to identify patients with CHF, based on 1 NACRS, DAD, SDS, or OHIP claim and a second claim (from either) in 1 year. The CHF database is limited to those aged 40 years or older.  OHIP: 428  DAD, SDS: ICD-9: 428, ICD-10: I500, I501, I509  Cardiac ischemic disease:  Any comorbidity in the past 5 years (DAD, any diagnosis field) or history of procedure in past 20 years (DAD, SDS), of the following:    Comorbidity (DAD, any diagnosis in the past 5 years):  Angina: ICD-10: I20    Chronic Ischemic Heart Disease: ICD-10: I25;  Myocardial infarction: ICD-10: I21, I22  Procedure (DAD & SDS):  Coronary Artery Bypass Grafting:  CCI procedure codes: 1IJ76  CCP procedure codes: 481    Percutaneous Coronary Intervention:  CCI procedure codes: 1IJ50, 1IJ54, 1IJ57GQ  CCP procedure codes: 4802, 4803  Atrial fibrillation:  Individuals with 1 hospitalization or 4 MD visits within a year in the past 5 years with the following codes:  ICD-9: 427.31, 427.32  ICD-10: I48  OHIP dxcode: 427 |
| Diabetes | ODD database | ≥1 hospitalizations [ICD 10 codes (E10-E14), ICD-9 (250)], or ≥2 OHIP claims [diagnosis code 250, Q040, K029, K030)] |
| Chronic respiratory disease | COPD database  ASTHMA database | ≥1 hospitalizations or ≥2 OHIP claims [ICD 10 codes (ICD 10: J41, J42, J43, J44, J45, J46)], [ICD 9 codes (491, 492, 493, 496)], and OHIP codes (140 to 209)  OR  ≥1 hospitalizations or ≥2 OHIP claims [ICD 10 codes (ICD 10: J45, J46)], [ICD 9 codes (493)] |
| Advanced liver disease (Cirrhosis or Decompensated Cirrhosis) | CIHI-DAD  OHIP | Defined using the Cirrhosis Algorithm:  Two or more physician visits (diagnosis code 571), or  one or more hospital diagnosis of cirrhosis, using the following diagnostic codes:  ICD-9: 456.1, 571.2, 571.5  ICD-10: I85.9, I98.2, K70.3, K71.7, K74.6  Defined using the Decompensated Cirrhosis Algorithm 5 (from above reference):  One or more physician visits with diagnosis code 571 and (one or more hospital diagnosis or one or more procedure), using the following diagnostic codes:  ICD-9: 456.0, 456.2, 572.2, 572.3, 572.4, 782.4, 789.5l; ICD-10: I85.0, I86.4, I98.20, I98.3, K721, K729, K76.6, K76.7, R17, R18  CCI: 1.NA.13.BA-FA, 1.NA.13.BA-X7, 1.NA.13.BA-BD, 1.KQ.76GP-NR, 1.OT.52.HA  CCP: 1006, 6691  OHIP: J057, Z591 |
| Dementia | CIHI-DAD  OHIP | Dementia (ICES cohort) definition:  1 hospitalization for dementia and/or 3 ambulatory visits for dementia, each separated by at least 30 days, within 2 years and/or 1 prescription from ODB.19 This variable was included a priori as hypothesized to be directly related to COVID-19 infection risk,3 as well as a marker for healthcare access, mobility, and household-level exposures.20,21  OHIP: 290, 331  DAD, SDS:  ICD-9: 0461, 290.0, 290.1, 290.2, 290.3, 290.4, 294, 331.0, 331.1, 331.5  ICD-10: F00, F01, F02, F03, G30  ODB:  1 prescription for a cholinesterase inhibitor |
| History of transient ischemic attack or acute ischemic stroke | CIHI-DAD  OHIP | This variable was included a priori as hypothesized to be directly related to COVID-19 infection risk.3  Transient Ischemic Attack:  DAD and NACRS were used to identify patients with a history of a transient ischemic attack, based on at least 1 hospitalization or ED visit with a diagnosis coded with one of the following codes:  ICD-9: 435, 3623  ICD-10: G450, G451, G452, G453, G458, G459, H340  Acute Ischemic Stroke:  DAD was used to identify patients with a history of acute ischemic stroke, based on at least 1 hospitalization with a main diagnosis coded with one of the following codes:  ICD-9: 434, 436; ICD-10: I63, I64, H34.1 |

**Supplementary Table S2. OHIP, ICD-9, and ICD-10 codes used to identify IBD-related health services utilization**

| Description | ICD-9 | OHIP code | ICD-10 |
| --- | --- | --- | --- |
| **IBD-specific** |  |  |  |
| Crohn's disease | 555.x | 555 | K50.x |
| Ulcerative colitis | 556.x | 556 | K51.x |
| **IBD-related** |  |  |  |
| Anorexia | 7830 | 787 | R63.0 |
| Abnormal Weight Gain | 783.1 |  | R63.5 |
| Abnormal Weight Loss | 783.2 |  | R63.4 |
| Lack of expected normal physiological development | 783.4 |  | R62.8, R62.9 |
| Symptoms involving digestive system, including: (787.0) Nausea and vomiting ; (787.1) Heartburn; (787.2) Dysphagia; (787.3) Gas/bloating; (787.6) Encopresis, fecal incontinence; (787.9) Other symptoms involving digestive system | 787.x | 787 | R11.x, R12.x, R13.x, R14.x, R15.x, R19.x |
| Abdominal pain | 7890 | 787 | R10.x |
| Dyspepsia | 536.8 | 536 | K30 |
| Cachexia | 799.4 |  | R64 |
| Esophagitis | 530.1 | 530 | K20, K21.x |
| Ulcer of esophagus | 5302 |  | K22.1 |
| Gastric ulcer | 531.x | 531 | K25.x |
| Duodenal ulcer | 532.x | 532 | K26.x |
| Peptic ulcer | 533.x |  | K27.x |
| GJ ulcer | 534.x | 534 | K28.x |
| Gastritis/duodenitis | 535.x | 535 | K29.x |
| Intestinal obstruction | 560.8  560.9 | 560 | K31.5  K56.6 |
| Rectal/anal hemorrhage | 569.3 | 569 | K62.5 |
| Other disorder of rectum/anus, including: ulcer; pain, sphincter tear (healed); dysplasia; other specified, including proctitis | 569.4 | 569 | K62.6, K62.8 |
| Abscess of the intestine | 569.5 | 569 | K63.0 |
| Other disorders of intestine, including: fistula (excl rectum); ulcer of intestine; perforation; angiodysplasia, no hemorrhage; angiodysplasia, with hemorrhage; dieulafoy; and other (including enteroptosis, granuloma of intestine, prolapse of intestine, pericolitis, perisigmoiditis, visceroptosis) | 569.8 | 569 | K63.2, K63.3, K63.1, K552.x, K638.x |
| Malabsorption | 262, 263.0, 263.1, 263.2, 263.9, 579.8, 579.9 | 579 | E43, E44.0, E44.1, E45, E46, K90.8, K90.9 |
| Anal Fistula | 565.1 | 565 | K60.3 |
| Anal Abscess | 566 | 566 | K610, K611, K612, K613, K614 |
| Ureteral Fistula | 593.8 |  | N288.1, N288.8 |
| Urethral Fistula | 599.1 |  | N36.0 |
| Fistula of stomach & duod | 537.4 |  | K31.6 |
| Vesical fistula | 596.2 |  | N32.2 |
| Fistula involving female GU | 619.x |  | N82.x |
| Hemorrhoids, including: (455.9) Anal skin tags | 455.x | 455 | I84.x |
| Rheumatoid arthritis | 713.1, 714.x, 716.4, 716.5, 716.6, 716.7, 716.8, 716.9 | 714 | M050, M052, M053, M058, M059, M060, M061 M062, M064, M068  M069, M070, M074, M075, M076, M080, M081, M082, M083, M084, M088, M089, M090, M091, M092, M098, M130, M131, M139 |
| Arthropathy associated GI cause | 713.3 |  | M074 M076 M075 |
| Inflammatory spondylopathies, including: (720.0) Ankylosing spondylitis; (720.1) Spinal enthesopathy; (720.2) Sacroiliitis; (720.8) Other inflammatory; (720.9) Other unspecified inflammatory | 720.x | 720 | M45.x, M46.x |
| Scleritis & episcleritis | 379.x | 379 | H15.x |
| Unspecified iridocyclitis (uveitis NOS) | 364.3 | 364 | H20.9 |
| Chorioretinitis, unspecified (uveitis, posterior NOS) | 363.2 | 363 | H30.9 |
| Acute and subacute iridocyclitis | 364 | 364 | H20.0 |
| Erythema nodosum | 695.2 | 695 | L52 |
| Pyoderma | 6860 | 686 | L08.0 |
| Pyogenic granuloma of the skin and soft tissue | 686.1 | 686 | L98.0 |
| Oral aphthae | 528.2 | 528 | K12.0 |
| Short stature | 783.4 |  | E34.3 |
| Osteoporosis | 7330.x, 7331 | 733 | M80.x, M81.x, M82.x, M83.x |
| Osteomyelitis | 730, 730.1, 730.2 | 730 | M86.x |
| Acute glomerulonephritis | 580.x | 580 | N00.x |
| Nephrolithiasis | 592.x | 592 | N20.x |
| Primary Sclerosing Cholangitis | 576.1 | 576 | K83.0 |
| Venous embolism/thrombosis | 453.x |  | I82.x |

**Supplementary Table S3. Sensitivity analyses of the SCCS method with risk windows extended to 42 days for serious AESIs, hospitalizations and ED visit events and 3-month risk window for gastroenterologist consultation visits after COVID-19 vaccination.**

| Outcome | Exposure/Risk Period | Number of Events | Cumulative PY | Crude Rate Per PY | Relative Incidence  Rate (95% CI) |  |
| --- | --- | --- | --- | --- | --- | --- |
| Adverse Event of Special Interest [within 42 days of dose] | Control period | 1572 | 80954.5 | 0.02 | Ref |  |
|  | Risk period after 1^st^ dose | 172 | 9895.9 | 0.02 | 0.89 (0.75-1.06) |  |
|  | Risk period after 2^nd^ dose | 218 | 10288.4 | 0.02 | 1.09 (0.92-1.29) |  |
|  | Risk period after 3^rd^ dose | 119 | 7845.1 | 0.02 | 0.79 (0.64-0.98) |  |
|  | Risk period after 4^th^ dose | 7 | 403.5 | 0.02 | 0.88 (0.31-2.47) |  |
| All Cause Hospitalization + ED Visits [within 42 days of dose] | Control period | 51817 | 80954.5 | 0.64 | Ref |  |
|  | Risk period after 1^st^ dose | 6242 | 9895.9 | 0.63 | 0.97 (0.94-1.01) |  |
|  | Risk period after 2^nd^ dose | 7042 | 10288.4 | 0.68 | 1.06 (1.03-1.10) |  |
|  | Risk period after 3^rd^ dose | 4115 | 7845.1 | 0.52 | 0.85 (0.82-0.88) |  |
|  | Risk period after 4^th^ dose | 243 | 403.5 | 0.60 | 0.85 (0.73-1.00) |  |
| All Cause Hospitalization [within 42 days of dose] | Control period | 12623 | 80954.5 | 0.16 | Ref |  |
|  | Risk period after 1^st^ dose | 1286 | 9895.9 | 0.13 | 0.83 (0.78-0.88) |  |
|  | Risk period after 2^nd^ dose | 1442 | 10288.4 | 0.14 | 0.90 (0.85-0.95) |  |
|  | Risk period after 3^rd^ dose | 974 | 7845.1 | 0.12 | 0.82 (0.76-0.88) |  |
|  | Risk period after 4^th^ dose | 63 | 403.5 | 0.16 | 0.82 (0.59-1.14) |  |
| All Cause ED visit [within 42 days of dose] | Control period | 47732 | 80954.5 | 0.59 | Ref |  |
|  | Risk period after 1^st^ dose | 5908 | 9895.9 | 0.60 | 1.00 (0.97-1.03) |  |
|  | Risk period after 2^nd^ dose | 6613 | 10288.4 | 0.64 | 1.08 (1.05-1.12) |  |
|  | Risk period after 3^rd^ dose | 3803 | 7845.1 | 0.48 | 0.85 (0.82-0.89) |  |
|  | Risk period after 4^th^ dose | 221 | 403.5 | 0.55 | 0.86 (0.73-1.01) |  |
| Gastroenterologist visit  [within 90 days] | Control period | 9890 | 58725.3 | 0.17 | Ref |  |
|  | Risk period after 1^st^ dose | 2280 | 15144.8 | 0.15 | 0.89 (0.84-0.94) |  |
|  | Risk period after 2^nd^ dose | 3513 | 21705.5 | 0.16 | 0.96 (0.92-1.00) |  |
|  | Risk period after 3^rd^ dose | 2500 | 15558.4 | 0.16 | 0.95 (0.91-1.00) |  |
|  | Risk period after 4^th^ dose | 156 | 554.2 | 0.28 | 1.41 (1.15-1.72) |  |
